# 3-(2,5-Di­methyl­phen­yl)-8-meth­oxy-2-oxo-1-aza­spiro­[4.5]dec-3-en-4-yl 3-(2-bromo-4-fluoro­phen­yl)acrylate

**DOI:** 10.1107/S160053681301430X

**Published:** 2013-06-08

**Authors:** Bing-Rong Xu, Xing-Rui He, Jing-Li Cheng, Jin-Hao Zhao

**Affiliations:** a College of Pharmaceutical Science, Zhejiang University of Technology, Hangzhou 310032, People’s Republic of China; b Ministry of Agriculture Key Laboratory of Agricultural Entomology, Institute of Pesticide and Environmental Toxicology, Zhejiang University, Hangzhou 310029, People’s Republic of China

## Abstract

In the title compound, C_27_H_27_BrFNO_4_, which is an inhibitor of acetyl-CoA carboxyl­ase, the cyclo­hexane ring displays a chair comformation with the spiro-C and meth­oxy-bearing C atoms deviating by 0.681 (7) and −0.655 (1) Å, resppectively, from the mean plane formed by the other four C atoms of the spiro-C_6_ ring. The mean planes of the cyclo­hexane and 2-bromo-4-fluoro­phenyl rings are nearly perpendicular to that of the pyrrolidine ring, making dihedral angles 89.75 (6) and 87.60 (9)°, respectively. In the crystal, mol­ecules are linked *via* pairs of N—H⋯O hydrogen bonds, forming inversion dimers.

## Related literature

For the pesticide spiro­tetra­mat (systematic name: cis-3-(2,5-di­methyl­phen­yl)-8-meth­oxy-2-oxo-1-aza­spiro­[4.5]dec-3-en-4-yl ethyl carbonate), the central unit of the title compound, see: Fischer & Weiss (2008 ▶); Maus (2008 ▶). For structures of spiro­tetra­mat derivatives, see: Fischer *et al.* (2010 ▶); Campbell *et al.* (1985 ▶); Schobert & Schlenk (2008 ▶); Zhao *et al.* (2012 ▶); Wang *et al.* (2011 ▶). For the metabolic transformation of spiro­tetra­mat, see: Bruck *et al.* (2009 ▶).
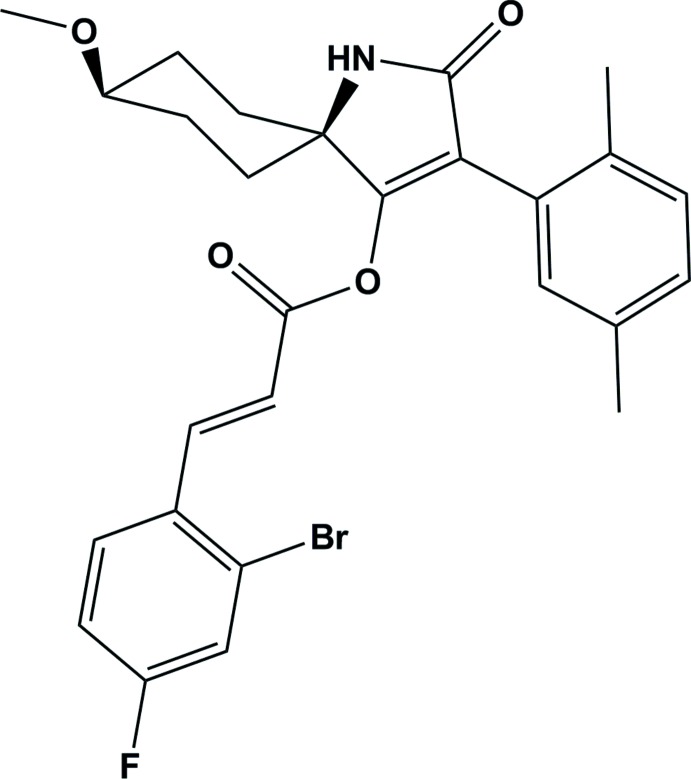


## Experimental

### 

#### Crystal data

C_27_H_27_BrFNO_4_
*M* *_r_* = 528.41
Triclinic, 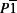

*a* = 10.5170 (5) Å
*b* = 11.2410 (6) Å
*c* = 12.5150 (7) Å
α = 110.364 (2)°
β = 102.049 (2)°
γ = 107.409 (1)°
*V* = 1239.95 (11) Å^3^
*Z* = 2
Mo *K*α radiation
μ = 1.70 mm^−1^
*T* = 296 K
0.48 × 0.45 × 0.24 mm

#### Data collection

Rigaku R-AXIS RAPID/ZJUG diffractometer
Absorption correction: multi-scan (*ABSCOR*; Higashi, 1995 ▶) *T*_min_ = 0.446, *T*_max_ = 0.665
10805 measured reflections
4845 independent reflections
3433 reflections with *I* > 2σ(*I*)
*R*_int_ = 0.035

#### Refinement

*R*[*F* ^2^ > 2σ(*F* ^2^)] = 0.041
*wR*(*F* ^2^) = 0.134
*S* = 1.00
4845 reflections
311 parameters
H-atom parameters constrained
Δρ_max_ = 0.46 e Å^−3^
Δρ_min_ = −0.95 e Å^−3^

### 

Data collection: *PROCESS-AUTO* (Rigaku, 2006 ▶); cell refinement: *PROCESS-AUTO*; data reduction: *CrystalStructure* (Rigaku, 2007 ▶); program(s) used to solve structure: *SHELXS97* (Sheldrick, 2008 ▶); program(s) used to refine structure: *SHELXL97* (Sheldrick, 2008 ▶); molecular graphics: *ORTEP-3 for Windows* (Farrugia, 2012 ▶); software used to prepare material for publication: *WinGX* (Farrugia, 2012 ▶).

## Supplementary Material

Crystal structure: contains datablock(s) global, I. DOI: 10.1107/S160053681301430X/vm2194sup1.cif

Structure factors: contains datablock(s) I. DOI: 10.1107/S160053681301430X/vm2194Isup2.hkl

Supplementary material file. DOI: 10.1107/S160053681301430X/vm2194Isup3.cdx

Supplementary material file. DOI: 10.1107/S160053681301430X/vm2194Isup4.cdx

Supplementary material file. DOI: 10.1107/S160053681301430X/vm2194Isup5.cml

Additional supplementary materials:  crystallographic information; 3D view; checkCIF report

## Figures and Tables

**Table 1:** Hydrogen-bond geometry (Å, °)

*D*—H⋯*A*	*D*—H	H⋯*A*	*D*⋯*A*	*D*—H⋯*A*
N1—H1⋯O1^i^	0.86	2.11	2.859 (4)	145

Symmetry code: (i) 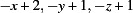.

**Fig. 1.**
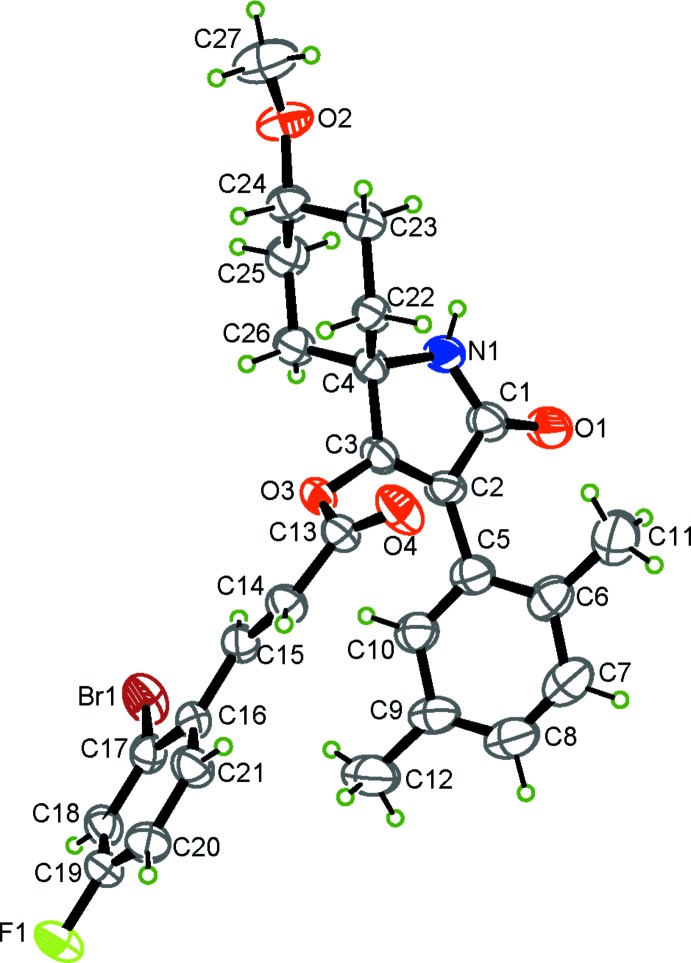
The molecular structure of (I), showing the atom-labelling scheme. Displacement ellipsoids are drawn at the 40% probability level.

**Fig. 2.**
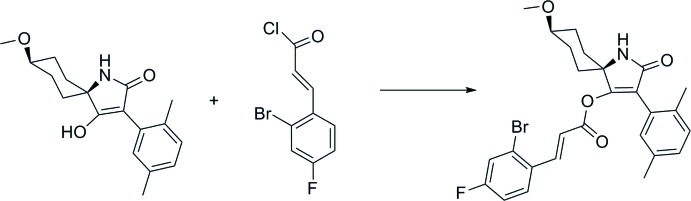
Reaction scheme.

## supplementary crystallographic information

### Comment

Spirotetramat is a new systemic insecticide which belongs chemically to the class of spirocyclic tetramic acid derivatives and was developed by Bayer CropScience AG (Fischer *et al.*, 2008; Maus, 2008). A unique mode of action coupled with a high degree of activity on targeted pests and low toxicity to nontarget organisms make spirocyclic tetronic acid compounds a new tool for integrated pest management (Bruck *et al.*, 2009; Campbell *et al.*,1985; Schobert *et al.*, 2008). Encouraged by previous papers from our laboratory (Zhao *et al.*, 2012; Wang *et al.*, 2011) in order to find the relationship between the ester at position C3 and biological activity, we synthesized the title compound by an esterification reaction and determined its molecular and crystal structure (Fig. 1).

The molecule contains two benzene rings, one six membered ring, and one five membered ring. The cyclohexane ring displays a chair conformation; atoms C22, C23, C25 and C26 lie in a plane with atoms C4 and C24 deviating by 0.681 (7) and -0.655 (1) Å. The mean plane of the cyclohexane ring and the benzene ring C16-C21 are nearly perpendicular to the pyrrolidine ring, making dihedral angles of 89.75 (6) and 87.60 (9) °, respectively. In the crystal, molecules are linked *via* a pair of N—H···O hydrogen bonds forming inversion dimers (Table 1).

### Experimental

The synthesis of the title compound is described in Fig. 2. In a flask, a solution of 3-(2-bromo-4-fluoro-phenyl)-acryloyl chloride (0.55 g, 2.0 mmoL) in anhydrous chloroform (5 ml) was added drop wise to a solution of *cis*-3-(2,5-dimethylphenyl)-4-hydroxy-8-methoxy-1-azaspiro[4.5]dec-3-en-2-one (0.40 g, 1.3 mmoL) in anhydrous chloroform (15 ml) at 0° C and stirred for 10 min. The reaction mixture was allowed to warm to room temperature and stirred at room temperature for 1 h. The reaction mixture was then washed with water (15 ml), saturated sodium bicarbonate (15 ml) and saturated sodium chloride solution and dried over anhydrous Na_2_SO_4_. The solvent was evaporated, and the residual solid was purified by flash column chromatography on silica gel using a mixture of petroleum ether (boiling point range 60–90° C) and ethyl acetate (4:1 by volume) as the eluent to give the title compound (0.33 g, 48%) as a colorless solid. The solid was filtrated and recrystallized with 95% ethanol to get colourless blocks. The ^1^H NMR, ESI-MS date testified the title compound's structure. ^1^H NMR (500 MHz, CDCl_3_): 8.02 (1*H*, d, J = 16 Hz, Ph—CH=CH–), 7.60–7.58 (1*H*, m, Ph—H), 7.40–7.38 (1*H*, m, Ph—H), 7.11–7.08 (2*H*, m, Ph—H), 7.04–7.02 (2*H*, m, Ph—H), 6.58 (1*H*, s, –NH–), 6.29 (1*H*, d, J = 16 Hz, Ph—CH=CH–), 3.40 (3*H*, s, –OCH_3_), 3.27–3.23 (1*H*, m, CH_3_OCH–), 2.28,2.26 (6*H*, s, Me_2_—Ar), 2.24–1.41 (8*H*, m, cyclohexane-H8); ESI-MS: 528 (*M*+H)^+^ (100%).

### Refinement

The H atoms were geometrically placed (C–H = 0.93–0.98 Å) and refined as riding with *U*_iso_(H) = 1.2*U*_eq_(C).

### Crystal data

**Table:**

C_27_H_27_BrFNO_4_	*Z* = 2
*M**_r_* = 528.41	*F*(000) = 544
Triclinic, *P*1	*D*_x_ = 1.415 Mg m^−^^3^
Hall symbol: -P 1	Mo *K*α radiation, λ = 0.71073 Å
*a* = 10.5170 (5) Å	Cell parameters from 8334 reflections
*b* = 11.2410 (6) Å	θ = 3.1–27.4°
*c* = 12.5150 (7) Å	µ = 1.70 mm^−^^1^
α = 110.364 (2)°	*T* = 296 K
β = 102.049 (2)°	Chunk, colorless
γ = 107.409 (1)°	0.48 × 0.45 × 0.24 mm
*V* = 1239.95 (11) Å^3^	

### Data collection

**Table:**

Rigaku R-AXIS RAPID/ZJUG diffractometer	4845 independent reflections
Radiation source: rotating anode	3433 reflections with *I* > 2σ(*I*)
Graphite monochromator	*R*_int_ = 0.035
Detector resolution: 10.00 pixels mm^-1^	θ_max_ = 26.0°, θ_min_ = 3.1°
ω scans	*h* = −12→12
Absorption correction: multi-scan (*ABSCOR*; Higashi, 1995)	*k* = −13→13
*T*_min_ = 0.446, *T*_max_ = 0.665	*l* = −15→15
10805 measured reflections	

### Refinement

**Table:**

Refinement on *F*^2^	Secondary atom site location: difference Fourier map
Least-squares matrix: full	Hydrogen site location: inferred from neighbouring sites
*R*[*F*^2^ > 2σ(*F*^2^)] = 0.041	H-atom parameters constrained
*wR*(*F*^2^) = 0.134	*w* = 1/[σ^2^(*F*_o_^2^) + (0.0452*P*)^2^ + 2.4533*P*] where *P* = (*F*_o_^2^ + 2*F*_c_^2^)/3
*S* = 1.00	(Δ/σ)_max_ < 0.001
4845 reflections	Δρ_max_ = 0.46 e Å^−^^3^
311 parameters	Δρ_min_ = −0.95 e Å^−^^3^
0 restraints	Extinction correction: *SHELXL97* (Sheldrick, 2008), Fc^*^=kFc[1+0.001xFc^2^λ^3^/sin(2θ)]^-1/4^
Primary atom site location: structure-invariant direct methods	Extinction coefficient: 0.036 (2)

### Special details

**Table:**

Geometry. All e.s.d.'s (except the e.s.d. in the dihedral angle between two l.s. planes) are estimated using the full covariance matrix. The cell e.s.d.'s are taken into account individually in the estimation of e.s.d.'s in distances, angles and torsion angles; correlations between e.s.d.'s in cell parameters are only used when they are defined by crystal symmetry. An approximate (isotropic) treatment of cell e.s.d.'s is used for estimating e.s.d.'s involving l.s. planes.
Refinement. Refinement of *F*^2^ against ALL reflections. The weighted *R*-factor *wR* and goodness of fit *S* are based on *F*^2^, conventional *R*-factors *R* are based on *F*, with *F* set to zero for negative *F*^2^. The threshold expression of *F*^2^ > σ(*F*^2^) is used only for calculating *R*-factors(gt) *etc*. and is not relevant to the choice of reflections for refinement. *R*-factors based on *F*^2^ are statistically about twice as large as those based on *F*, and *R*- factors based on ALL data will be even larger.

### Fractional atomic coordinates and isotropic or equivalent isotropic displacement parameters (Å^2^)

**Table:**

	*x*	*y*	*z*	*U*_iso_*/*U*_eq_	
C1	0.8088 (4)	0.4745 (4)	0.4082 (4)	0.0461 (9)	
C2	0.6693 (4)	0.4235 (4)	0.3104 (4)	0.0427 (9)	
C3	0.5971 (4)	0.2940 (4)	0.2902 (3)	0.0383 (8)	
C4	0.6755 (4)	0.2431 (4)	0.3664 (3)	0.0368 (8)	
C5	0.6237 (4)	0.5058 (4)	0.2510 (4)	0.0478 (10)	
C6	0.7037 (5)	0.5696 (4)	0.1946 (4)	0.0517 (10)	
C7	0.6475 (6)	0.6406 (5)	0.1396 (4)	0.0634 (13)	
H7	0.6991	0.6852	0.1026	0.076*	
C8	0.5196 (6)	0.6467 (5)	0.1384 (4)	0.0648 (13)	
H8	0.4867	0.6957	0.1012	0.078*	
C9	0.4375 (5)	0.5814 (4)	0.1916 (4)	0.0538 (11)	
C10	0.4938 (4)	0.5121 (4)	0.2489 (4)	0.0489 (10)	
H10	0.4422	0.4689	0.2868	0.059*	
C11	0.8437 (6)	0.5624 (6)	0.1891 (6)	0.0758 (15)	
H11A	0.9195	0.6344	0.2621	0.114*	
H11B	0.8595	0.5745	0.1200	0.114*	
H11C	0.8408	0.4736	0.1816	0.114*	
C12	0.2922 (5)	0.5783 (6)	0.1840 (5)	0.0744 (15)	
H12A	0.2922	0.6668	0.1937	0.112*	
H12B	0.2672	0.5584	0.2473	0.112*	
H12C	0.2241	0.5075	0.1063	0.112*	
C13	0.4365 (4)	0.1713 (4)	0.0883 (3)	0.0392 (8)	
C14	0.2895 (4)	0.1370 (4)	0.0199 (3)	0.0389 (8)	
H14	0.2559	0.0845	−0.0640	0.047*	
C15	0.2014 (4)	0.1765 (4)	0.0705 (3)	0.0368 (8)	
H15	0.2330	0.2179	0.1551	0.044*	
C16	0.0597 (4)	0.1620 (4)	0.0080 (3)	0.0368 (8)	
C17	−0.0245 (4)	0.2068 (4)	0.0720 (3)	0.0375 (8)	
C18	−0.1587 (4)	0.1930 (4)	0.0134 (4)	0.0433 (9)	
H18	−0.2139	0.2221	0.0571	0.052*	
C19	−0.2076 (4)	0.1352 (4)	−0.1108 (4)	0.0450 (9)	
C20	−0.1310 (4)	0.0910 (4)	−0.1794 (4)	0.0498 (10)	
H20	−0.1671	0.0532	−0.2639	0.060*	
C21	0.0027 (4)	0.1043 (4)	−0.1186 (4)	0.0448 (9)	
H21	0.0561	0.0738	−0.1637	0.054*	
C22	0.7009 (4)	0.1194 (4)	0.2883 (3)	0.0369 (8)	
H22A	0.6100	0.0438	0.2336	0.044*	
H22B	0.7531	0.1460	0.2391	0.044*	
C23	0.7841 (4)	0.0692 (4)	0.3654 (3)	0.0416 (9)	
H23A	0.7944	−0.0113	0.3124	0.050*	
H23B	0.8785	0.1417	0.4148	0.050*	
C24	0.7083 (4)	0.0313 (4)	0.4473 (3)	0.0422 (9)	
H24	0.6171	−0.0484	0.3967	0.051*	
C25	0.6811 (4)	0.1511 (4)	0.5247 (4)	0.0478 (10)	
H25A	0.7713	0.2267	0.5811	0.057*	
H25B	0.6272	0.1222	0.5720	0.057*	
C26	0.5993 (4)	0.2028 (4)	0.4484 (4)	0.0446 (9)	
H26A	0.5887	0.2827	0.5019	0.054*	
H26B	0.5050	0.1305	0.3983	0.054*	
C27	0.7981 (6)	−0.1319 (5)	0.4762 (5)	0.0770 (16)	
H27A	0.7041	−0.2030	0.4282	0.115*	
H27B	0.8414	−0.1527	0.5390	0.115*	
H27C	0.8543	−0.1281	0.4251	0.115*	
N1	0.8079 (3)	0.3695 (3)	0.4373 (3)	0.0448 (8)	
H1	0.8779	0.3758	0.4920	0.054*	
O1	0.9056 (3)	0.5907 (3)	0.4548 (3)	0.0610 (9)	
O2	0.7899 (3)	−0.0026 (3)	0.5295 (3)	0.0570 (8)	
O3	0.4596 (2)	0.2081 (3)	0.2103 (2)	0.0393 (6)	
O4	0.5294 (3)	0.1721 (3)	0.0476 (3)	0.0579 (8)	
F1	−0.3399 (2)	0.1198 (3)	−0.1697 (3)	0.0642 (7)	
Br1	0.04050 (4)	0.28752 (5)	0.24507 (4)	0.0578 (2)	

### Atomic displacement parameters (Å^2^)

**Table:**

	*U*^11^	*U*^22^	*U*^33^	*U*^12^	*U*^13^	*U*^23^
C1	0.037 (2)	0.041 (2)	0.049 (2)	0.0123 (17)	0.0039 (17)	0.0168 (19)
C2	0.036 (2)	0.042 (2)	0.043 (2)	0.0138 (16)	0.0015 (16)	0.0204 (18)
C3	0.0278 (17)	0.041 (2)	0.039 (2)	0.0122 (15)	0.0059 (14)	0.0151 (17)
C4	0.0283 (18)	0.0381 (19)	0.037 (2)	0.0086 (14)	0.0044 (14)	0.0163 (16)
C5	0.045 (2)	0.037 (2)	0.044 (2)	0.0096 (16)	−0.0010 (17)	0.0142 (18)
C6	0.060 (3)	0.040 (2)	0.048 (2)	0.0157 (19)	0.014 (2)	0.0195 (19)
C7	0.080 (3)	0.050 (3)	0.054 (3)	0.018 (2)	0.016 (2)	0.028 (2)
C8	0.075 (3)	0.049 (3)	0.059 (3)	0.023 (2)	0.002 (2)	0.026 (2)
C9	0.060 (3)	0.042 (2)	0.047 (2)	0.021 (2)	−0.001 (2)	0.016 (2)
C10	0.048 (2)	0.040 (2)	0.050 (2)	0.0134 (17)	0.0057 (18)	0.0190 (19)
C11	0.076 (4)	0.077 (4)	0.091 (4)	0.031 (3)	0.042 (3)	0.047 (3)
C12	0.071 (3)	0.067 (3)	0.082 (4)	0.037 (3)	0.007 (3)	0.033 (3)
C13	0.0295 (19)	0.041 (2)	0.042 (2)	0.0128 (15)	0.0072 (15)	0.0181 (17)
C14	0.0355 (19)	0.041 (2)	0.0323 (19)	0.0132 (15)	0.0068 (15)	0.0132 (16)
C15	0.0328 (18)	0.043 (2)	0.0289 (18)	0.0108 (15)	0.0071 (14)	0.0157 (16)
C16	0.0298 (18)	0.0363 (19)	0.041 (2)	0.0090 (14)	0.0090 (14)	0.0187 (16)
C17	0.0363 (19)	0.0359 (19)	0.036 (2)	0.0109 (15)	0.0118 (15)	0.0151 (16)
C18	0.035 (2)	0.041 (2)	0.057 (3)	0.0176 (16)	0.0194 (17)	0.0208 (19)
C19	0.0293 (19)	0.047 (2)	0.059 (3)	0.0141 (16)	0.0085 (17)	0.029 (2)
C20	0.043 (2)	0.058 (3)	0.042 (2)	0.0150 (19)	0.0043 (17)	0.026 (2)
C21	0.036 (2)	0.055 (2)	0.039 (2)	0.0165 (17)	0.0084 (16)	0.0203 (19)
C22	0.0361 (19)	0.040 (2)	0.0355 (19)	0.0161 (15)	0.0134 (15)	0.0172 (16)
C23	0.041 (2)	0.044 (2)	0.040 (2)	0.0190 (17)	0.0126 (16)	0.0176 (17)
C24	0.046 (2)	0.046 (2)	0.033 (2)	0.0185 (17)	0.0075 (16)	0.0181 (17)
C25	0.053 (2)	0.055 (2)	0.040 (2)	0.0229 (19)	0.0172 (18)	0.0237 (19)
C26	0.043 (2)	0.052 (2)	0.044 (2)	0.0228 (18)	0.0163 (17)	0.0226 (19)
C27	0.109 (4)	0.055 (3)	0.068 (3)	0.046 (3)	0.015 (3)	0.027 (3)
N1	0.0353 (17)	0.0384 (17)	0.0474 (19)	0.0095 (13)	−0.0018 (14)	0.0183 (15)
O1	0.0406 (16)	0.0398 (16)	0.076 (2)	0.0051 (13)	−0.0066 (14)	0.0217 (15)
O2	0.080 (2)	0.0511 (17)	0.0417 (16)	0.0353 (16)	0.0093 (14)	0.0224 (14)
O3	0.0250 (12)	0.0469 (15)	0.0410 (14)	0.0099 (10)	0.0051 (10)	0.0214 (12)
O4	0.0389 (16)	0.082 (2)	0.0533 (18)	0.0284 (15)	0.0198 (13)	0.0247 (16)
F1	0.0347 (12)	0.0731 (17)	0.0796 (18)	0.0215 (11)	0.0030 (11)	0.0380 (15)
Br1	0.0467 (3)	0.0738 (4)	0.0435 (3)	0.0204 (2)	0.01813 (19)	0.0176 (2)

### Geometric parameters (Å, º)

**Table:**

C1—O1	1.228 (5)	C15—C16	1.463 (5)
C1—N1	1.350 (5)	C15—H15	0.9300
C1—C2	1.496 (5)	C16—C21	1.395 (5)
C2—C3	1.327 (5)	C16—C17	1.401 (5)
C2—C5	1.494 (5)	C17—C18	1.383 (5)
C3—O3	1.383 (4)	C17—Br1	1.903 (4)
C3—C4	1.500 (5)	C18—C19	1.367 (6)
C4—N1	1.473 (4)	C18—H18	0.9300
C4—C26	1.533 (5)	C19—F1	1.359 (4)
C4—C22	1.532 (5)	C19—C20	1.366 (6)
C5—C10	1.385 (6)	C20—C21	1.388 (5)
C5—C6	1.400 (6)	C20—H20	0.9300
C6—C7	1.398 (6)	C21—H21	0.9300
C6—C11	1.513 (7)	C22—C23	1.529 (5)
C7—C8	1.364 (7)	C22—H22A	0.9700
C7—H7	0.9300	C22—H22B	0.9700
C8—C9	1.389 (7)	C23—C24	1.520 (5)
C8—H8	0.9300	C23—H23A	0.9700
C9—C10	1.399 (6)	C23—H23B	0.9700
C9—C12	1.501 (7)	C24—O2	1.429 (4)
C10—H10	0.9300	C24—C25	1.508 (6)
C11—H11A	0.9600	C24—H24	0.9800
C11—H11B	0.9600	C25—C26	1.527 (5)
C11—H11C	0.9600	C25—H25A	0.9700
C12—H12A	0.9600	C25—H25B	0.9700
C12—H12B	0.9600	C26—H26A	0.9700
C12—H12C	0.9600	C26—H26B	0.9700
C13—O4	1.192 (4)	C27—O2	1.410 (5)
C13—O3	1.381 (5)	C27—H27A	0.9600
C13—C14	1.461 (5)	C27—H27B	0.9600
C14—C15	1.324 (5)	C27—H27C	0.9600
C14—H14	0.9300	N1—H1	0.8600
			
O1—C1—N1	126.2 (4)	C18—C17—C16	122.0 (3)
O1—C1—C2	126.7 (4)	C18—C17—Br1	116.8 (3)
N1—C1—C2	107.2 (3)	C16—C17—Br1	121.1 (3)
C3—C2—C5	128.4 (3)	C19—C18—C17	117.8 (3)
C3—C2—C1	106.0 (3)	C19—C18—H18	121.1
C5—C2—C1	125.6 (3)	C17—C18—H18	121.1
C2—C3—O3	126.1 (3)	F1—C19—C20	118.0 (4)
C2—C3—C4	114.4 (3)	F1—C19—C18	118.5 (4)
O3—C3—C4	119.5 (3)	C20—C19—C18	123.5 (3)
N1—C4—C3	99.0 (3)	C19—C20—C21	117.6 (4)
N1—C4—C26	111.7 (3)	C19—C20—H20	121.2
C3—C4—C26	112.8 (3)	C21—C20—H20	121.2
N1—C4—C22	112.4 (3)	C20—C21—C16	122.2 (4)
C3—C4—C22	111.8 (3)	C20—C21—H21	118.9
C26—C4—C22	108.9 (3)	C16—C21—H21	118.9
C10—C5—C6	120.2 (4)	C23—C22—C4	112.2 (3)
C10—C5—C2	117.0 (4)	C23—C22—H22A	109.2
C6—C5—C2	122.7 (4)	C4—C22—H22A	109.2
C7—C6—C5	117.0 (4)	C23—C22—H22B	109.2
C7—C6—C11	119.8 (4)	C4—C22—H22B	109.2
C5—C6—C11	123.2 (4)	H22A—C22—H22B	107.9
C8—C7—C6	122.3 (5)	C24—C23—C22	110.8 (3)
C8—C7—H7	118.8	C24—C23—H23A	109.5
C6—C7—H7	118.8	C22—C23—H23A	109.5
C7—C8—C9	121.4 (4)	C24—C23—H23B	109.5
C7—C8—H8	119.3	C22—C23—H23B	109.5
C9—C8—H8	119.3	H23A—C23—H23B	108.1
C8—C9—C10	116.9 (4)	O2—C24—C25	106.3 (3)
C8—C9—C12	122.4 (4)	O2—C24—C23	112.4 (3)
C10—C9—C12	120.7 (4)	C25—C24—C23	110.9 (3)
C5—C10—C9	122.2 (4)	O2—C24—H24	109.0
C5—C10—H10	118.9	C25—C24—H24	109.0
C9—C10—H10	118.9	C23—C24—H24	109.0
C6—C11—H11A	109.5	C24—C25—C26	112.1 (3)
C6—C11—H11B	109.5	C24—C25—H25A	109.2
H11A—C11—H11B	109.5	C26—C25—H25A	109.2
C6—C11—H11C	109.5	C24—C25—H25B	109.2
H11A—C11—H11C	109.5	C26—C25—H25B	109.2
H11B—C11—H11C	109.5	H25A—C25—H25B	107.9
C9—C12—H12A	109.5	C25—C26—C4	111.7 (3)
C9—C12—H12B	109.5	C25—C26—H26A	109.3
H12A—C12—H12B	109.5	C4—C26—H26A	109.3
C9—C12—H12C	109.5	C25—C26—H26B	109.3
H12A—C12—H12C	109.5	C4—C26—H26B	109.3
H12B—C12—H12C	109.5	H26A—C26—H26B	107.9
O4—C13—O3	122.2 (3)	O2—C27—H27A	109.5
O4—C13—C14	125.7 (4)	O2—C27—H27B	109.5
O3—C13—C14	112.1 (3)	H27A—C27—H27B	109.5
C15—C14—C13	123.8 (3)	O2—C27—H27C	109.5
C15—C14—H14	118.1	H27A—C27—H27C	109.5
C13—C14—H14	118.1	H27B—C27—H27C	109.5
C14—C15—C16	127.1 (3)	C1—N1—C4	113.4 (3)
C14—C15—H15	116.4	C1—N1—H1	123.3
C16—C15—H15	116.4	C4—N1—H1	123.3
C21—C16—C17	116.8 (3)	C27—O2—C24	115.2 (3)
C21—C16—C15	121.4 (3)	C13—O3—C3	116.7 (3)
C17—C16—C15	121.8 (3)		
			
O1—C1—C2—C3	−178.7 (4)	C21—C16—C17—C18	0.9 (5)
N1—C1—C2—C3	0.4 (5)	C15—C16—C17—C18	−179.8 (3)
O1—C1—C2—C5	0.7 (7)	C21—C16—C17—Br1	−180.0 (3)
N1—C1—C2—C5	179.8 (4)	C15—C16—C17—Br1	−0.7 (5)
C5—C2—C3—O3	−2.3 (7)	C16—C17—C18—C19	−0.8 (6)
C1—C2—C3—O3	177.1 (4)	Br1—C17—C18—C19	−180.0 (3)
C5—C2—C3—C4	179.9 (4)	C17—C18—C19—F1	179.4 (3)
C1—C2—C3—C4	−0.8 (5)	C17—C18—C19—C20	−0.1 (6)
C2—C3—C4—N1	0.8 (4)	F1—C19—C20—C21	−178.6 (3)
O3—C3—C4—N1	−177.2 (3)	C18—C19—C20—C21	0.9 (6)
C2—C3—C4—C26	119.1 (4)	C19—C20—C21—C16	−0.8 (6)
O3—C3—C4—C26	−58.9 (4)	C17—C16—C21—C20	−0.1 (6)
C2—C3—C4—C22	−117.7 (4)	C15—C16—C21—C20	−179.4 (4)
O3—C3—C4—C22	64.2 (4)	N1—C4—C22—C23	68.2 (4)
C3—C2—C5—C10	51.7 (6)	C3—C4—C22—C23	178.5 (3)
C1—C2—C5—C10	−127.5 (4)	C26—C4—C22—C23	−56.1 (4)
C3—C2—C5—C6	−125.6 (5)	C4—C22—C23—C24	56.8 (4)
C1—C2—C5—C6	55.2 (6)	C22—C23—C24—O2	−174.0 (3)
C10—C5—C6—C7	1.2 (6)	C22—C23—C24—C25	−55.1 (4)
C2—C5—C6—C7	178.4 (4)	O2—C24—C25—C26	177.6 (3)
C10—C5—C6—C11	−177.6 (4)	C23—C24—C25—C26	55.1 (4)
C2—C5—C6—C11	−0.4 (7)	C24—C25—C26—C4	−55.9 (5)
C5—C6—C7—C8	−1.0 (7)	N1—C4—C26—C25	−69.6 (4)
C11—C6—C7—C8	177.9 (5)	C3—C4—C26—C25	179.9 (3)
C6—C7—C8—C9	−0.4 (8)	C22—C4—C26—C25	55.1 (4)
C7—C8—C9—C10	1.5 (7)	O1—C1—N1—C4	179.2 (4)
C7—C8—C9—C12	−175.7 (5)	C2—C1—N1—C4	0.1 (5)
C6—C5—C10—C9	−0.1 (6)	C3—C4—N1—C1	−0.5 (4)
C2—C5—C10—C9	−177.4 (4)	C26—C4—N1—C1	−119.6 (4)
C8—C9—C10—C5	−1.3 (6)	C22—C4—N1—C1	117.6 (4)
C12—C9—C10—C5	176.0 (4)	C25—C24—O2—C27	165.5 (4)
O4—C13—C14—C15	−158.5 (4)	C23—C24—O2—C27	−73.0 (5)
O3—C13—C14—C15	19.6 (5)	O4—C13—O3—C3	23.9 (5)
C13—C14—C15—C16	171.6 (3)	C14—C13—O3—C3	−154.3 (3)
C14—C15—C16—C21	−1.4 (6)	C2—C3—O3—C13	64.2 (5)
C14—C15—C16—C17	179.3 (4)	C4—C3—O3—C13	−118.0 (4)

### Hydrogen-bond geometry (Å, º)

**Table:**

*D*—H···*A*	*D*—H	H···*A*	*D*···*A*	*D*—H···*A*
N1—H1···O1^i^	0.86	2.11	2.859 (4)	145

Symmetry code: (i) −*x*+2, −*y*+1, −*z*+1.
